# Evaluation of tolerance towards ureteral double-J-stents in children: an unmet need

**DOI:** 10.1007/s00431-025-06165-x

**Published:** 2025-05-27

**Authors:** Dafni Planta, Raphael N. Vuille-dit-Bille, Stefan Holland-Cunz, Martina Frech-Doerfler

**Affiliations:** Division of Pediatric Surgery, University Children’s Hospital Basel, University of Basel, Spitalstrasse 33, 4056 Basel, Switzerland

**Keywords:** Ureteral stent, Quality of life, Lower urinary tract symptom, Pain, Complication

## Abstract

Ureteric double-J-stents (DJS) are commonly applied devices in urology that often cause irritative symptoms in adults, leading to decreased quality of life. Little is known about how they are tolerated by children. Furthermore, standardized patient-centered care for children has not yet been established. The objective of this observational study is to assess the tolerance of children towards DJS in relation to their age by evaluating their irritative symptom rate and to raise awareness of the need for a standardized evaluation method. A prospective observational single-center study was conducted over a period of 14 months on children with indwelling DJS. A questionnaire addressing 5 categories (voiding symptoms, general symptoms, social activities and/or sexual health, catheter removal, and medication) was developed for different age groups and employed after stent removal. Twenty patients with a mean age of 5.8 years (range 3 months to 15 years) were enrolled in the study. During the indwelling time (mean 6 weeks), 13 patients suffered from irritative symptoms (intermittent pain and/or voiding problems). There was a positive association between age and the reported pain score (Spearman’s ρ = 0.54, *p* = 0.04). Overall, the quality of life (QoL) was less impaired than in adult patients and the complications were less severe, but the symptom occurrence rate was similarly high.

*Conclusion*: The current study lays the groundwork for a standardized re-evaluation of ureteral stents in pediatric applications. Through the development of a specialized questionnaire, we provide a quantitative tool for assessing the QoL of children with indwelling ureteral stents. The next step in standardizing the use of DJS in the pediatric population involves the validation of our questionnaire through a large-scale multi-center study.

**Table:** 

**What is Known:** *• Ureteric double-J stents are commonly used devices in pediatric urology.* • *Double-J stents are often not well tolerated by adults due to severe irritating symptoms, which decreases their quality of life.*	
**What is New:** *• In children, little is known about tolerance of double-J stents.* • *There is no standardized method to assess tolerance in children, especially with regard to age.*

**What is Known:**

*• Ureteric double-J stents are commonly used devices in pediatric urology.*

• *Double-J stents are often not well tolerated by adults due to severe irritating symptoms, which decreases their quality of life.*

**What is New:**

*• In children, little is known about tolerance of double-J stents.*

• *There is no standardized method to assess tolerance in children, especially with regard to age.*

## Introduction

In pediatric urology, stenting of the ureter or drainage of the renal pelvis are often necessary due to obstruction or after urological interventions. Beside transcutaneous catheters (e.g., pyeloplasty catheters), ureteral double-J-stents (DJS) are applied for various reasons, including the reduction of risk for postoperative strictures and urinary leakage into the abdominal cavity [1, 2]. In pediatric urology, DJS are mainly applied during pyeloplasties and ureteral reimplantations [2–4], although stentless surgery is an option.

Other applications include stenting for conservative treatment of congenital megaureter, after ureteral trauma, or after extracorporeal shock wave lithotripsy [5–7]. Typically, DJS are left indwelling for several weeks and are then removed cystoscopically [4, 6, 8, 9].

Indwelling DJS cause irritative symptoms in up to 80% of adult patients, thereby decreasing their quality of life (QoL) [10–12]. Patients often report voiding problems such as urge symptoms or radiating pain in the suprapubic region, flanks, or genitalia [4, 11]. This has led to the development of new stent designs and the use of drugs aimed at reducing stent-related irritative symptoms [13–15]. In 2003, Joshi et al. developed the Ureteral Stent Symptom Questionnaire (USSQ), which categorizes the morbidity caused by DJS into 5 areas: voiding problems, pain, sexual health, work life, and general health [16]. Since its development, the USSQ has been an established tool for evaluating the stent-related quality of life in adults [16].

In pediatric urology, neither a comparable questionnaire exists nor have stent-related irritative symptoms been adequately studied yet. There is no standardized method to assess various stent designs and drugs aimed at reducing stent-related irritative symptoms. This study aims to evaluate a novel age-adapted questionnaire to assess the tolerance of ureteric DJS in children, adapted from the USSQ. Furthermore, our goal was to evaluate irritative and other symptoms in children following DJS insertion using this novel questionnaire.

## Materials and methods

We conducted a prospective observational single-center study between December 2020 and February 2022 including all consecutive patients treated with uni- or bilateral DJS. The inserted DJS were either conventional non-magnetic stents (Cook Medical) or magnetic stents (mDJS) (Magnetic Blackstar® Stent, Urotech-Urovision GmbH). The decision about which stent to use was made intraoperatively by the pediatric urologist and following the patient’s and/or caregiver’s preferred removal method. The patients received their first outpatient follow-up 1 week postoperatively and then every 3 to 4 weeks until the DJS was removed. All patients received antibiotic prophylaxis with trimethoprim/sulfamethoxazole as long as DJS were in place. The study was approved by the local ethics committee (reference number: 2020–00948), and written informed consent was obtained from all patients and/or caregivers. The following factors were assessed: age, gender, diagnosis, surgical procedure, DJS indwelling time, medication, complications, and discomfort during the indwelling time as well as during the removal of the stent. A questionnaire was developed based on the USSQ. The questionnaire was adapted to fit five age groups: (1) toddlers (0–1 year), (2) infants (2–5 years), (3) school children (6–10 years), (4) teenagers 1 (11–15 years), and (5) teenagers 2 (16–18 years). In all age groups, the questionnaire addresses voiding problems, pain, medication, and, in case of an mDJS, the removal of the stent including parents’ satisfaction. For age groups 3, 4, and 5, the questionnaire additionally evaluates everyday school life, and for group 5 sexual health and sexual activity. Depending on the patient’s age, pain was assessed using the Visual Analog Scale (for children aged 11–18) [17], the Faces Pain Scale-Revised (for children aged 5–10) [18], or the KUSS-Scale (for children aged 0–4) [19]. After stent removal, the questionnaire was filled out by either the caregiver (groups 1 and 2), the patient (groups 4 and 5), or both (group 3) during the consultation with the attending pediatric urologist. Conventional DJS were removed cystoscopically under general anesthesia and the removal of mDJS was conducted in an outpatient setting during consultation. In case of magnetic stent removal, the pre-emptive administration of analgesics and/or anxiolytics was determined in consultation with the caregivers and/or the patient. Depending on the age and patient, medication consisted of Midazolam (oral or rectal), Fentanyl (nasal), and/or N_2_O (inhalative). Furthermore, in male patients, transabdominal bedside sonography was conducted during the retrieval procedure to visualize the connection between the two magnets.

## Statistical analysis

Statistical analysis was performed using GraphPad Prism 8.0 (GraphPad Software, La Jolla, California) by Spearman’s correlation test for analysis of association between age and reported pain score, Fisher’s exact test for comparison of the irritative symptom rate as well as the rate of urinary tract infections (UTI) between patients with conventional DJS and mDJS, and unpaired *t*-test for mean age comparison between groups with and without irritative symptoms as well as mean pain score of patients with conventional DJS and mDJS. Statistically significant variances were defined as *p* < 0.05.

## Results

Between December 2020 and February 2022, 22 patients were treated with uni- or bilateral DJS, 20 of whom accepted to participate in the study. None were lost to follow-up. The median age was 2.5 years (range 3 months–15 years) (Table 1).

**Table 1:** Patient’s data

Patient’s data	*n* = 20 patients
Gender [m/f] (%)	16/4 (80%/20%)
Age [years] (median + minimum–maximum)	2.5 (3 months–15 years)
Stent in place [weeks] (median + minimum–maximum)	4.5 (2–24)
Surgical Indication (%)	
- Ureteropelvic junction obstruction (UJO) - UJO due to crossing accessory renal vessel - Refluxive-obstructive Megaureter - Vesico ureteral junction obstruction - Nephrolithiasis - Traumatic injury to the ureter - Kidney failure (initially urethral valves)	8 (40%) 4 (20%) 3 (15%) 2 (10%) 1 (5%) 1 (5%) 1 (5%)
Type of surgery (%)	
- Laparoscopic pyeloplasty - Ureterocystoneostomy (Politano-Leadbetter) - Open pyeloplasty - DJS placement only - Percutaneous nephrolithotripsy	10 (50%) 5 (25%) 2 (10%) 2 (10%) 1 (5%)
Type of stent	
- Magnetic - Conventional	15 (75%) 5 (25%)
Stent size (mean + range)	
- Length [cm] - Diameter [Fr]	15.3 (8–26) 4.9 (3.7–8)

Sixteen patients (80%) were male and 4 (20%) were female. The median DJS indwelling time was 4.5 weeks (range 2–24 weeks). The most common indication for DJS insertion was laparoscopic pyeloplasty (*n* = 10, 50%), followed by Politano-Leadbetter ureteral reimplantation (*n* = 5, 25%) (Table 1). All patients received antibiotic prophylaxis with trimethoprim/sulfamethoxazole throughout the indwelling time.

**Fig. 1:**
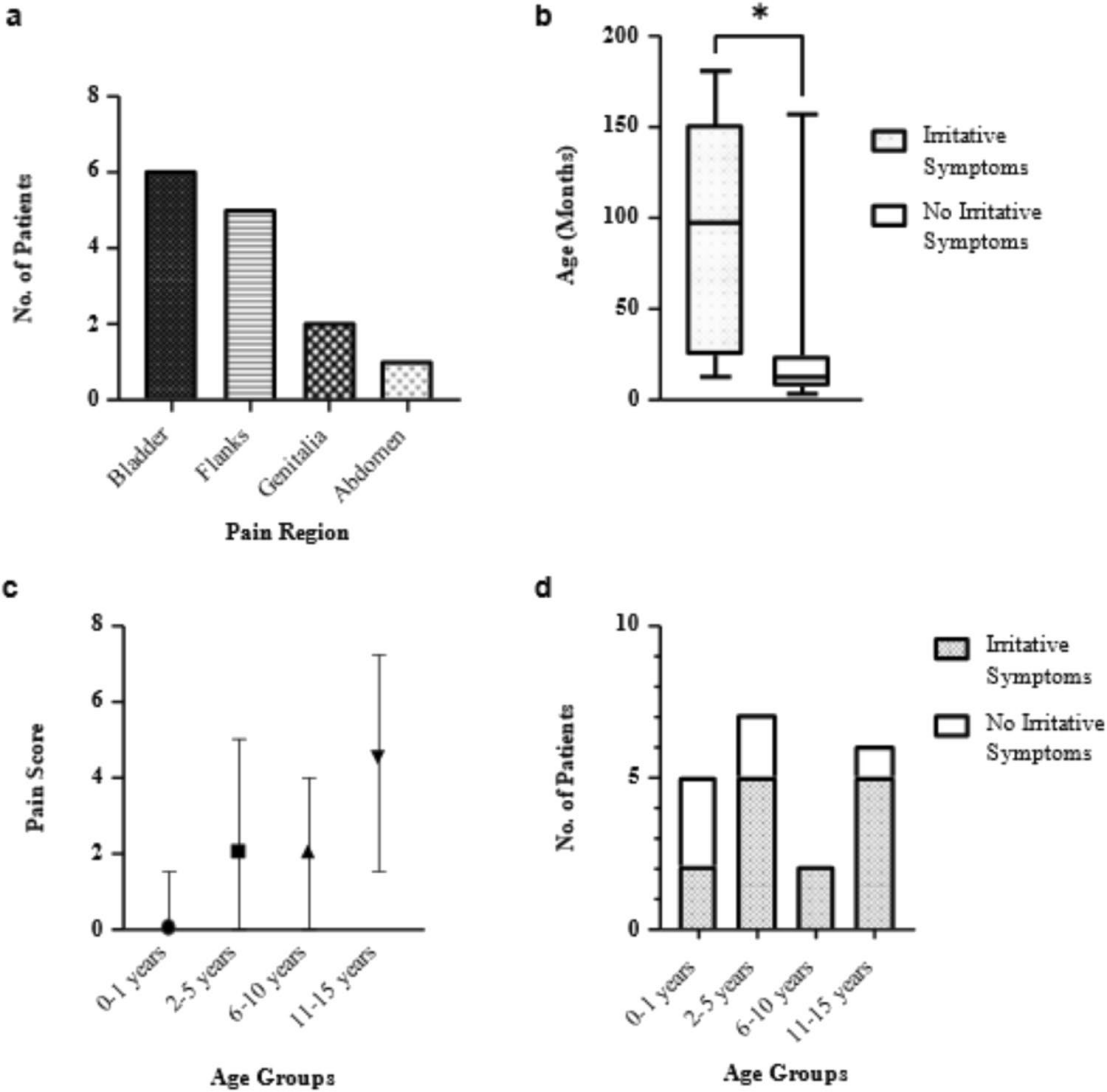
Stent-related irritative symptoms. **a** Pain regions reported by the patients during the stent indwelling time, **b** the median age of patients experiencing irritative symptoms and of patients without irritative symptoms (data are median, 95% CI, and minimum–maximum), **c** pain score experienced in each age group during stent indwelling time (data are median and 95% CI), **d** the number of patients experiencing irritative symptoms and no irritative symptoms within each age group

Thirteen patients (65%) suffered from intermittent pain/agitation and/or voiding problems during the indwelling time and the bladder was the most frequent locus of pain (Fig. 1a). In five patients, anticholinergic treatment was started within the first week after stent insertion due to suspected bladder cramps.

In the age group 2 and upwards, 3 patients (20%) only had pain during physical activity, 8 patients (53%) additionally experienced pain at rest, and 1 patient (7%) only had pain during micturition (Table 2). In the age group 3 and upwards, 2 patients (25%) reported additional voiding problems, such as urge symptoms, involuntary loss of urine, and increased voiding frequency (Table 2).

**Table 2:** Outcomes

General symptoms	
*0–18 years*	*n*** = 20**
- Pain/agitation and/or pain during micturition	13 (65%)
*0–1 years*	*n*** = 5**
- Pain	1 (20%)
- Agitation	1 (20%)
*2–18 years*	*n*** = ***15*
- Pain	11 (73%)
- Situation during which pain occurred	
- Only during physical activity	3 (20%)
- During physical activity and at rest	8 (53%)
- Only during micturition	1 (7%)
- Pain locus	
Bladder	6 (40%)
Flanks	5 (33%)
Genitalia	2 (13%)
Abdomen	1 (6%)
**Pain Score** (median + minimum–maximum)	
All age groups (0–18 years) (*n* = 20)	2 (0–8/10)
Age group 1 (0–1 years) (*n* = 5)	0 (0–3/10)
Age group 2 (2–5 years) (*n* = 7)	2 (0–8/10)
Age group 3 (6–10 years) (*n* = 2)	2 (0–5/10)
Age group 4 (11–15 years) (*n* = 6)	4.5 (0–8)
**Voiding problems**	
*0–18 years*	*n*** = ***20*
- Hematuria [weeks] (mean + minimum–maximum)	2.5 (1–16)
- Analgesic intake [days] (mean + minimum–maximum)	14 (1–120)
*6–18 years*	*n*** = ***8*
- Urge symptoms	0
- Nocturnal enuresis	2 (25%)
- Increased voiding frequency	2 (25%)
- Involuntary loss of urine	1 (13%)
**Social activities**	
*6–18 years*	*n*** = ***8*
- No physical activities > 4 weeks postoperatively	1 (13%)
- School absence > 1 week postoperatively	1 (13%)

The median pain score during the indwelling time for age group 1, 2, 3, and 4 was 0 (0–3/10), 2 (0–8/10), 2 (0–5/10), and 4.5 (0–8/10), respectively (Table 2). Statistical analyses showed a significant positive association between age and pain score with a high effect according to Cohen (Spearman’s ρ = 0.54, *p* = 0.04). Patients that reported pain and/or voiding problems during the indwelling time were older than those without (*M* = 90.46 vs. 33.86, *R*^2^ = 0.18) (Fig. 1b–d).

Six patients (30%) had premature stent removal due to either irritative symptoms (15%) (3, 4, and 5 weeks after stent insertion) or febrile UTI (15%) (10 days, 3 weeks, and 4 weeks after stent insertion, caused by *E. coli*, *Klebsiella pneumoniae*, and *Enterococcus faecalis*, respectively). Following pain and voiding problems, persistent hematuria and UTIs were the third and fourth most common complications, respectively. Two further complications consisted of one proximal stent dislocation and one obstructed stent. Overall, 16 patients (80%) showed complications and/or symptoms caused by the indwelling DJS (pain, voiding problems, persistent hematuria, stent migration, UTI, and obstructed stent). According to the grading system for surgical complications of Clavien-Dindo, 83.3% of complications were grade I, 12.5% were grade II and 4.1% were grade IIIb (Table 3).

**Table 3:** Severity grade of complications according to Clavien-Dindo

Complication	*n*	Grade according to Clavien-Dindo
Irritative symptoms	13	I
Persistent hematuria > 2 weeks	6	I
Stent migration	1	I
UTI	3	II
Obstructed stent	1	IIIb
Total	**24**	

**Fig. 2:**
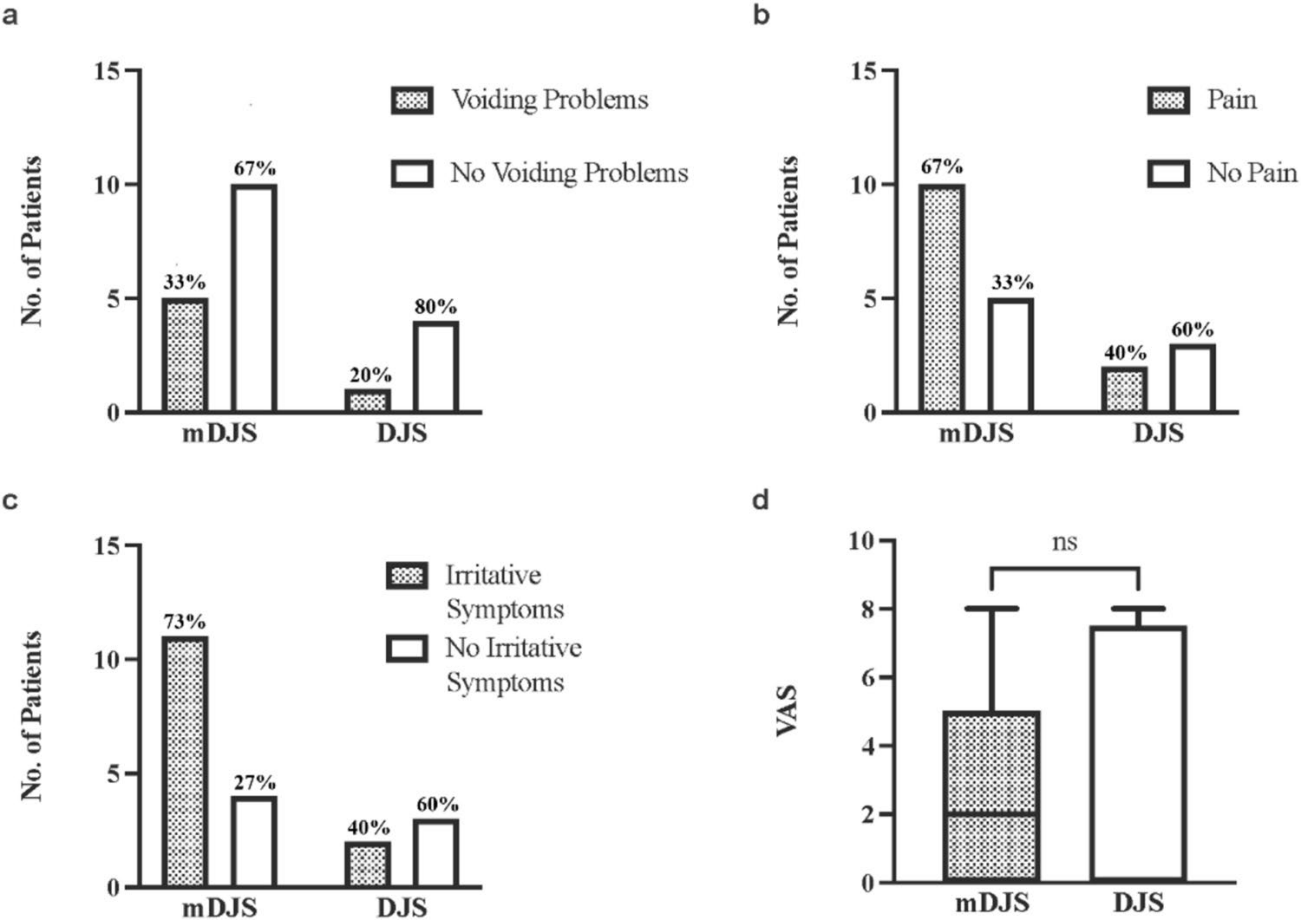
Magnetic DJS versus conventional DJS. **a** Number of patients experiencing voiding problems and no voiding problems compared between magnetic double-J-stent (mDJS) and conventional double-J-stent (DJS). **b** Number of patients experiencing pain and no pain compared between mDJS and DJS. **c** Number of patients experiencing irritative symptoms and no irritative symptoms between mDJS and DJS. **d** VAS (visual analog scale) pain score experienced by patients with mDJS and DJS, scores from the Faces Pain Scale-Revised (for children aged 5–10) or the KUS-Scale (for children aged 0–4) were converted to VAS (data are median, 95% CI, and minimum–maximum)

Fifteen patients (75%) had a magnetic DJS. All of them could be retrieved in an outpatient setting without general anesthesia. The use of an mDJS showed a tendency towards a higher association of voiding problems (OR 2, 95% CI 0.18–29) (Fig. 2a) and pain during the indwelling time (OR 3, 95% CI 0.46–20) (Fig. 2b), as well as a higher overall risk of irritative symptoms (OR 4.1, 95% CI 0.6–28) (Fig. 2c). However, none of the above were statistically significant. Given pain, there was no significant difference in pain score between the mDJS and the DJS group (*p* = 0.8) (Fig. 2d). The use of an mDJS was not associated with a higher risk of UTI (OR 0.62, CI 0.06 to 10.96).

## Discussion

Our questionnaire enabled a sensitive assessment of the tolerance and morbidity in children with indwelling DJS. We reported an irritative symptom rate as high as 65%, emphasizing the need for standardized monitoring and management of this patient group.

Due to the low incidence of conditions requiring DJS insertions in children, our study enrolled a small number of patients. The ureteropelvic junction obstruction and refluxive-obstructive megaureter, which were responsible for the majority of inserted stents in our patients, have an incidence of 1:1000–1:4000 in newborns and only 10–15% of the cases require surgery [3]. We recruited patients over a period of 14 months in one of the nine training centers for pediatric surgery in Switzerland with a participation rate of 90%. To achieve higher statistical power, a much longer recruiting period or a multi-center study is needed. The questionnaire used in this study is not validated yet; however, it was adapted from the USSQ [16]. Hereby, our goal was to assess the irritative symptom rate in children with DJS and prove the need for a validated questionnaire, which should be the next step in standardizing the use of DJS in the pediatric population. The majority of our patients received mDJS, as their feasibility in children has been proven in multiple recent studies [4, 20–22]. Compared to children treated with conventional stents, patients in our cohort with mDJS had more irritative symptoms and similar pain rates, indicating that mDJS may be a confounding factor in our study.

Sixty-five percent of our patients reported irritative symptoms consisting of pain and/or voiding problems. The symptom rate increased with age; however, an observer bias cannot be excluded as with decreasing age the evaluation of subjective symptoms becomes less accurate and we had to rely on the caregivers’ assessment. Within the schoolchildren and teenager group of our study, none of the patients were sexually active yet and only one child showed intermittent school absence > 1 week postoperatively. The median pain score of 4.5/10 recorded in age group 4 corresponds to moderate to moderately strong pain [18]. According to Clavien-Dindo, out of an overall complication rate of 80%, we recorded 83.3% grade I complications, 12.5% grade II, and 4.1% grade IIIb. While hematuria did not affect patients’ well-being, UTIs increased morbidity and led to premature stent removal. In our study, 3 out of 20 patients (15%) experienced a UTI. Our results show the safe feasibility of mDJS in children, but also a higher probability for patients with an indwelling mDJS to develop irritative symptoms.

Joshi et al. conducted two prospective studies on patients with indwelling ureteral stents in 2001 and 2002, enrolling 120 and 48 patients, respectively [10, 11]. In both studies, 80% of the patients reported stent-related pain and bothersome urinary symptoms such as storage symptoms, incontinence, symptoms of dysuria, and hematuria [10, 11]. The patients also reported reduced work performance, reduced working hours, and sexual dysfunction, altogether impairing their QoL significantly [11]. Geavlete et al. retrospectively evaluated the complications on 50′000 DJS insertions in adults from 1996 to 2021 and recorded an overall complication rate of 82% (*n* = 41,369) [12]. According to the grading system for surgical complications by Clavien-Dindo [21], 47.1% of complications were grade I, 44.8% grade II, and 7.9% grade IIIa (the last 0.2% accounted for grade IIIB-V) [12]. Approximately 50% (*n* = 25′979) of the patients suffered from irritative voiding symptoms and lumbar pain [12].

Few studies have assessed complications of indwelling DJS in children and none have evaluated irritative symptoms as a primary objective so far. Awad et al., Castagnetti et al., and Teklali et al., all of whom evaluated the role of DJS in primary obstructive megaureter, reported overall complication rates of 40%, 70%, and 34.2%, respectively [6, 8, 9]. The complications included stent encrustation, stent migration, UTI, persistent intermittent hematuria, renal pelvis abscess, and stent nodes. Pain and voiding problems were mentioned briefly but were not described in detail. Ben-Meir et al. and Garcia-Aparicio et al. assessed UTI occurrence in 82 and 67 children with DJS, respectively, reporting UTI rates of 9.75% [23] and of 5.9% [24], respectively.

In children, the stent extraction under cystoscopic guidance typically requires general anesthesia, which bears potential negative effects, especially in toddlers and infants [25]. Moreover, cystoscopic stent removal in general anesthesia is related to higher personnel expenses, higher overall costs, and a higher rate of complications compared to non-cystoscopic stent removals [26]. Stentless surgery (for example during ureteral reimplantations), or the use of transcutaneous catheters (as, e.g., used in pyeloplasties), reflect alternative treatment options with the advantage of not causing irritative symptoms and avoidance of secondary anesthesia. Alternatively, the pursuit of a stent extraction method without the need for cystoscopy has led to a rising interest in the Magnetic Blackstar Stent in Pediatric Urology, which is reflected in the number of studies published on this topic in the last few years [4, 20–22]. The stent has an integrated distal magnetic tip and can be extracted with a magnetic retrieval device consisting of a 7 or 9 Fr urethral catheter. The consent regarding the QoL in relation to the use of mDJS in adults remains inconclusive: Farouk et al. showed a significantly higher score in the USSQ in patients with mDJS [20] whereas Diranzo-Garcia et al. showed no difference between the two groups [21] and Rassweiler et al. merely showed a difference in the pain localization [22]. No studies evaluating irritative mDJS-related symptoms in children exist up to date. An alternative for avoiding a cystoscopy for stent removal is the attachment of an extraction string, which affixes the distal loop to the inner thighs or labia majora; however, it bears the risk of a distal dislocation [26–28]. Other methods grouped under the term “snare technique” consist of an inexpensive alternative to mDJS and describe the retrieval of the DJS with a thread loop that is inserted through the urethra via a feeding tube [29]. Compared to magnetic retrieval, only a few studies have evaluated the feasibility of extraction strings and the snare technique in children with DJS.

Compared to the literature, our questionnaire allowed a sensitive assessment of irritative symptoms related to indwelling DJS. The irritative symptom rate in our study is similar to that reported in the adult population [10–12, 16]. The daily life activities of pediatric patients < 15 years of age are less impaired than the ones of adults and the complications were of lesser severity, bearing in mind that a stent re-insertion in children needs general anesthesia and thus corresponds to a complication grade IIIb according to Dindo and Clavien [30]. Our study shows a slightly higher UTI rate (15%) than what is reported in the literature (5.9–9.75%) [23, 24]. One way of minimizing stent complications including infection rate is the reduction of the indwelling time. Abdelwahab et al., who conducted a prospective randomized trial with 36 pediatric patients with laparoscopic pyeloplasties, compared a stent indwelling time of 1 week versus 4 weeks and showed no difference in the outcome one month postoperatively [31]. None of the patients in our study suffered from an infection within the first postoperative week, which supports Abdelwahab et al.’s suggestion of removing DJS after 1 week. We, therefore, advocate for shortened stent indwelling times. In summary, our study revealed a high irritative symptom rate in children with DJS, emphasizing the necessity for adopting age-dependent treatment protocols.

## Conclusion

The current study lays the groundwork for a standardized re-evaluation of ureteral stents in pediatric applications. Through the development of a specialized questionnaire, we provide a quantitative tool for assessing the QoL of children with indwelling ureteral stents. Our results indicate that children with indwelling DJS experience a high rate of irritative symptoms, which increases with age. The questionnaire allows for the evaluation of drugs aiming to reduce stent-related symptoms and the evaluation of new stent designs, such as the mDJS, for each age group. To achieve patient-centered care in clinical practice, the high occurrence rate of irritative symptoms must be considered. The next step in standardizing the use of DJS in the pediatric population involves the validation of our questionnaire through a large-scale multi-center study.

## Data Availability

The data that support the findings of this study are available from the corresponding author, M.F., upon reasonable request.

## Abbreviations

DJS: Double-j-stent
mDJS: Magnetic double-j-stent
USSQ: Ureteral stent symptom questionnaire
UTI: Urinary tract infection
QoL: Quality of life

## References

[1] Siggers JH, Waters S, Wattis J, Cummings L (2009) Flow dynamics in a stented ureter. Math Med Biol 26(1):1–24. 10.1093/imammb/dqn020 18990681 10.1093/imammb/dqn020

[2] Gearhart JP, Rink RC, Mouriquand PDE (2010) Pediatric urology, 2nd edn. Elsevier, Philadelphia

[3] Heinrich M, Neuhaus K (2012) Kinderchirurgie Basiswissen und Praxis. In: Schweinitz Dv (ed) Kinderchirurgie Basiswissen und Praxis. 2. W. Zuckschwerdt Verlag GmbH, Germering, p S. 173-5, S. 9-83, S. 87-97

[4] Brillat Arce W, Vuille-Dit-Bille RN, Holland-Cunz SG, Frech-Doerfler M (2021) Magnetic double-J-stent removal without general anaesthesia in children. Urology 156:251–255. 10.1016/j.urology.2021.01.028 33493511 10.1016/j.urology.2021.01.028

[5] Shen P, Jiang M, Yang J, Li X, Li Y, Wei W et al (2011) Use of ureteral stent in extracorporeal shock wave lithotripsy for upper urinary calculi: a systematic review and meta-analysis. J Urol 186(4):1328–1335. 10.1016/j.juro.2011.05.073 21855945 10.1016/j.juro.2011.05.073

[6] Castagnetti M, Cimador M, Sergio M, De Grazia E (2006) Double-J stent insertion across vesicoureteral junction–is it a valuable initial approach in neonates and infants with severe primary nonrefluxing megaureter? Urology 68(4):870–5; discussion 5-6. 10.1016/j.urology.2006.05.052 17070371 10.1016/j.urology.2006.05.052

[7] Guerriero WG (1989) Ureteral injury. Urol Clin North Am 16(2):237–248 2711544

[8] Teklali Y, Robert Y, Boillot B, Overs C, Piolat C, Rabattu PY (2018) Endoscopic management of primary obstructive megaureter in pediatrics. J Pediatr Urol 14(5):382–387. 10.1016/j.jpurol.2018.05.027 30006257 10.1016/j.jpurol.2018.05.027

[9] Awad K, Woodward MN, Shalaby MS (2019) Long-term outcome of JJ stent insertion for primary obstructive megaureter in children. J Pediatr Urol 15(1):66.e1-.e5. 10.1016/j.jpurol.2018.09.011 10.1016/j.jpurol.2018.09.011 30385050

[10] Joshi HB, Stainthorpe A, Keeley FX, MacDonagh R, Timoney AG (2001) Indwelling ureteral stents: evaluation of quality of life to aid outcome analysis. J Endourol 15(2):151–154. 10.1089/089277901750134421 11325084 10.1089/089277901750134421

[11] Joshi HB, Okeke A, Newns N, Keeley FX, Timoney AG (2002) Characterization of urinary symptoms in patients with ureteral stents. Urology 59(4):511–516. 10.1016/s0090-4295(01)01644-2 11927301 10.1016/s0090-4295(01)01644-2

[12] Geavlete P, Georgescu D, Mulțescu R, Stanescu F, Cozma C, Geavlete B (2021) Ureteral stent complications - experience on 50,000 procedures. J Med Life 14(6):769–75. 10.25122/jml-2021-0352 35126746 10.25122/jml-2021-0352 PMC8811679

[13] Chew BH, Lange D (2016) Advances in ureteral stent development. Curr Opin Urol 26(3):277–282. 10.1097/MOU.0000000000000275 26840739 10.1097/MOU.0000000000000275

[14] Tharwat M, Elsaadany MM, Lashin AM, El-Nahas AR (2018) A randomized controlled trial evaluating sildenafil citrate in relieving ureteral stent-related symptoms. World J Urol 36(11):1877–1881. 10.1007/s00345-018-2339-y 29766318 10.1007/s00345-018-2339-y

[15] Tae BS, Cho S, Jeon BJ, Choi H, Park JY, Cho SY et al (2018) Does mirabegron relieve ureteric stent-related discomfort? A prospective, randomized, multicentre study. BJU Int 122(5):866–872. 10.1111/bju.14416 29802800 10.1111/bju.14416

[16] Joshi HB, Newns N, Stainthorpe A, MacDonagh RP, Keeley FX, Timoney AG (2003) Ureteral stent symptom questionnaire: development and validation of a multidimensional quality of life measure. J Urol 169(3):1060–1064. 10.1097/01.ju.0000049198.53424.1d 12576846 10.1097/01.ju.0000049198.53424.1d

[17] Collins SL, Moore RA, McQuay HJ (1997) The visual analogue pain intensity scale: what is moderate pain in millimetres? Pain 72(1–2):95–97. 10.1016/s0304-3959(97)00005-5 9272792 10.1016/s0304-3959(97)00005-5

[18] Hicks CL, von Baeyer CL, Spafford PA, van Korlaar I, Goodenough B (2001) The faces pain scale-revised: toward a common metric in pediatric pain measurement. Pain 93(2):173–183. 10.1016/S0304-3959(01)00314-1 11427329 10.1016/S0304-3959(01)00314-1

[19] Büttner W, Finke W, Hilleke M, Reckert S, Vsianska L, Brambrink AM (1998) Entwicklung eines Fremdbeobachtungsbogens zur Beurteilung des postoperativen Schmerzes bei Säuglingen. Anasthesiol Intensivmed Notfallmed Schmerzther 33:353–361 9689392 10.1055/s-2007-994263

[20] Farouk A, Tawfick A, Hasan M, Abuftira AA, Maged WA (2019) Can magnitip double-J stent serve as a substitute for a standard double-J stent? Turk J Urol 45(6):437–443. 10.5152/tud.2019.19038 31603418 10.5152/tud.2019.19038 PMC6788565

[21] Diranzo-Garcia M, Pardo-Duarte P, Álvarez-Barrera A, Juan-Escudero JU, Beltrán-Puig M, Monzó-Cataluña A et al (2021) Magnetic double-J stent: evaluation of tolerance and impact on quality of life compared to traditional double-J stent. Actas Urol Esp (Engl Ed) 45(5):366–372. 10.1016/j.acuroe.2021.04.004 34088436 10.1016/j.acuroe.2021.04.004

[22] Rassweiler MC, Michel MS, Ritter M, Honeck P (2017) Magnetic ureteral stent removal without cystoscopy: a randomized controlled trial. J Endourol 31(8):762–766. 10.1089/end.2017.0051 28478732 10.1089/end.2017.0051

[23] Ben-Meir D, Golan S, Ehrlich Y, Livne PM (2009) Characteristics and clinical significance of bacterial colonization of ureteral double-J stents in children. J Pediatr Urol 5(5):355–358. 10.1016/j.jpurol.2009.01.001 19251483 10.1016/j.jpurol.2009.01.001

[24] García-Aparicio L, Blázquez-Gómez E, Martin O, Krauel L, de Haro I, Rodó J (2015) Bacterial characteristics and clinical significance of ureteral double-J stents in children. Actas Urol Esp 39(1):53–56. 10.1016/j.acuro.2014.04.008 24954842 10.1016/j.acuro.2014.04.008

[25] McCann ME, Soriano SG (2019) Does general anesthesia affect neurodevelopment in infants and children? BMJ 367:l6459. 10.1136/bmj.l6459 31818811 10.1136/bmj.l6459

[26] Juliebø-Jones P, Pietropaolo A, Haugland JN, Mykoniatis I, Somani BK (2022) Current status of ureteric stents on extraction strings and other non-cystoscopic removal methods in the paediatric setting: a systematic review on behalf of the European Association of Urology (EAU) Young Academic Urology (YAU) Urolithiasis Group. Urology 160:10–16. 10.1016/j.urology.2021.11.022 34910924 10.1016/j.urology.2021.11.022

[27] Oliver R, Wells H, Traxer O, Knoll T, Aboumarzouk O, Biyani CS et al (2018) Ureteric stents on extraction strings: a systematic review of literature. Urolithiasis 46(2):129–136. 10.1007/s00240-016-0898-1 27324264 10.1007/s00240-016-0898-1 PMC5852195

[28] Kajbafzadeh AM, Nabavizadeh B, Keihani S, Hosseini Sharifi SH (2015) Revisiting the tethered ureteral stents in children: a novel modification. Int Urol Nephrol 47(6):881–885. 10.1007/s11255-015-0963-7 25838032 10.1007/s11255-015-0963-7

[29] Sundaramurthy S, Joseph Thomas R, Herle K, Jeyaseelan Mathai J, Jacob Kurian J (2019) Double J stent removal in paediatric patients by Vellore Catheter Snare technique: a randomised control trial. J Pediatr Urol 15(6):661.e1-.e8. 10.1016/j.jpurol.2019.08.009 10.1016/j.jpurol.2019.08.009 31586540

[30] Dindo D, Demartines N, Clavien PA (2004) Classification of surgical complications: a new proposal with evaluation in a cohort of 6336 patients and results of a survey. Ann Surg 240(2):205–213. 10.1097/01.sla.0000133083.54934.ae 15273542 10.1097/01.sla.0000133083.54934.ae PMC1360123

[31] Abdelwahab M, Abdelaziz A, Aboulela W, Shouman AM, Ghoneima W, Shoukry A et al (2020) One week stenting after pediatric laparoscopic pyeloplasty; is it enough? J Pediatr Urol 16(1):98.e1-.e6. 10.1016/j.jpurol.2019.10.016 10.1016/j.jpurol.2019.10.016 31786228

